# Framing artificial intelligence in Chilean digital press before and after the launch of ChatGPT: From concern to optimism

**DOI:** 10.1371/journal.pone.0348680

**Published:** 2026-05-11

**Authors:** Ana Bucchi, Carola Neira-Mellado, Rubén Sanchez-Sabate, Manuel Mora-Chepo

**Affiliations:** 1 Departamento de Odontología Integral Adultos, Facultad de Odontología, Universidad de La Frontera, Temuco, Chile; 2 Núcleo Científico Tecnológico en Biorecursos (BIOREN), Universidad de La Frontera, Temuco, Chile; 3 Doctorado en Comunicación, Universidad de La Frontera, Temuco, Chile - Universidad Austral de Chile, Valdivia, Chile; 4 Núcleo en Ciencias Sociales y Humanidades, Universidad de La Frontera, Temuco, Chile; 5 Núcleo de Investigación en Educación, Ciencias Sociales y Patrimonio, Universidad Adventista de Chile, Chillán, Chile; 6 Facultad de Educación, Ciencias Sociales y Humanidades, Universidad de La Frontera, Temuco, Chile; University of Basel Institute for Biomedical Ethics: Universitat Basel Institut fur Bio- und Medizinethik, SWITZERLAND

## Abstract

This article examines the evolution of AI framing in Chilean national digital media before and after the launch of ChatGPT in 2022. Through topic modeling and emotion analysis of 1,466 articles published by six media outlets over periods that begin between 2000 and 2014, depending on the outlet, and extend in all cases until 2024, this study identifies significant thematic and affective changes. In the pre-ChatGPT period, coverage combined recognition of AI’s capabilities with concerns regarding labor displacement, governance, and human identity, frequently referencing global corporations and state institutions. In the post-ChatGPT period, topics became narrower in scope, with fewer actors and greater emphasis on universities and cultural organizations implementing AI. The focus shifted from the development of AI systems to their applications, predominantly framed in positive terms. At the same time, negative sentiment was largely confined to epistemological uncertainty. This transformation aligns with national surveys indicating growing public optimism, diverges from Global North debates centered on regulation and privacy, and converges with East Asian framings that are oriented toward innovation and creativity. These findings suggest that the launch of ChatGPT coincided with a reinforcement of a socio-technical imaginary of AI as inevitable and beneficial, yet potentially limiting the diversity of public debate.

## Introduction

The acceptance and implementation of AI are influenced not only by its technical efficiency but also by stories that resonate with local values, expectations, and concerns. Media coverage plays a central role in this process, as it shapes public understanding and frames societal responses to emerging technologies. However, such coverage varies markedly across countries and regions, reflecting distinct political, cultural, and economic contexts [1,2].

In this paper, we examine how the digital press in Chile mediates public perceptions and discussions about AI. Chile is a case of interest because public attitudes towards AI have changed rapidly. While skepticism toward AI’s long-term benefits was relatively high until recently [3], the growing adoption of AI tools in daily life has coincided with a rising share of the population viewing AI as an opportunity rather than a threat [4].

This study presents the first comprehensive framing analysis of AI news across the majority of Chile’s high-traffic digital outlets that combines topic modeling, entity identification, and emotion analysis by topic. By expanding the range of news sources examined, it significantly advances prior research on Chilean AI media coverage [5] and deepens understanding of how the press shapes public consensus, highlights specific AI attributes, and positions diverse perspectives within the national discourse. Our findings indicate that Chile exhibits a distinctive configuration of dominant topics that shifted significantly since the launch of ChatGPT. This evolution reflects a growing convergence in Chilean news discourse toward emphasizing AI’s positive and concrete aspects, while critical and risk-focused viewpoints remain present but less prominent. Notably, this pattern aligns Chile more closely with some Eastern media representations, such as those in China, diverging from Western media’s stronger focus on individual risks.

## Theoretical framework

### Sociology of technology and technological change

This study is informed by sociological perspectives that view technological change as a process of mutual shaping between society and technology [6,7]. These approaches are particularly relevant for understanding how public discourse about AI evolves, as they provide conceptual tools for analyzing the negotiations, alliances, and future-oriented visions that shape technological trajectories. This is vividly illustrated in the Chilean context, where the state has actively intervened to shape the nation’s AI trajectory. Strategic initiatives, such as the 2021 National AI Policy, the creation of the National AI Center (CENIA), significant public investment in AI doctoral scholarships, and the deployment of 5G infrastructure, have positioned Chile as a regional leader, topping the Latin American AI Index (ILIA) in 2024 [8]. These efforts represent a concerted attempt to influence the interpretive flexibility of AI by aligning its meaning with national development, economic competitiveness, and technological modernization.

Survey data indicate a complex landscape of public engagement with AI. Chile reports one of the highest rates of AI use worldwide [3], and 8–9 out of 10 people are aware of the technology [4]. However, more than a third of the population does not use AI in their daily lives, and distrust remains a key barrier; skepticism toward AI companies is also widespread. A notable portion of the population also expressed concerns about inequality, privacy, and the use of biometric data. These patterns suggest that public perceptions of AI are diverse, ambivalent, and potentially at odds with official narratives.

In this context, the sociology of technology provides an ideal interpretive framework for understanding the configuration of AI as an emerging technology. The foundation of this approach lies in rejecting a linear view of technology, which assumes that it is implemented exactly as designed by experts [6]. Instead, this perspective argues that technology is shaped by diverse social groups, including experts, political actors, and civil society, who participate in what is known as sociotechnical controversies. Thus, technology does not exist as such but is instead constructed by the social groups that interact with it.

There are two major approaches in the sociology of technology. On the one hand, the Social Construction of Technology (SCOT) framework conceptualizes such diversity of perspectives as central to technological change. Different social groups interpret technologies in divergent ways, and stabilization or closure occurs not because one version is technically superior but because certain specific regulations—often backed by legal, economic, or symbolic resources—prevail [6,9]. From this perspective, the Chilean state’s innovation-centric strategy can be seen as a macro-level attempt to promote a particular understanding of AI and to shape the broader field of negotiation.

In contrast, Actor–Actor–Network Theory (ANT) complements this perspective by focusing less on predefined social groups and more on the shifting configurations of actors and alliances that emerge during a controversy. It expands the analysis to include both human and non-human actants, recognizing their capacity to influence outcomes [10,11]. From this standpoint, a technology’s success depends on the ability to translate different interests and enroll a heterogeneous network that sustains its development [12,13].

Over the past two decades, the sociology of technology has been enriched by literature on expectations and sociotechnical imaginaries [14,15]. Expectations are shared visions of desirable futures that orient innovation, while imaginaries embed such visions in broader cultural narratives about progress and social order. In Chile, state strategies and public perceptions together illustrate the dynamic and sometimes divergent imaginaries surrounding AI: while official narratives emphasize modernization and competitiveness, survey data point to enduring doubts, skepticism, and uneven adoption of AI [3,4].

Sociotechnical expectations and imaginaries complement the sociology of technology perspective by incorporating contextual elements into the development of a controversy while also establishing an interpretive framework that connects such outcomes with the broader development of the society in which they unfold. They emphasize both the social construction of technology and its influence on public opinion, as well as the creation of institutions and regulatory frameworks.

Together, these perspectives provide a multidimensional lens for analyzing how technologies are negotiated, stabilized, and projected into the future of the field. This approach is particularly relevant for AI, whose opacity, rapid evolution, and ongoing debates make it a fertile ground for various interpretations, expectations, and concerns.

### Framing analysis

The news media are a fundamental agent in shaping the meanings and uses of new technologies. First, the media function as gatekeepers, as the “obligatory passage point” for news flow and public engagement in industrialized societies. Second, by producing and circulating the news, the media decide which actors and their respective interests will reach citizens’ attention. Third, the general public has hardly any other source of knowledge on such foreign topics as new and disruptive technologies. Therefore, the news media have the power to shape public discourse on AI by defining expectations around it, as well as by gradually or rapidly building, news by news, a sociotechnical imaginary about AI.

Media effects theories, such as Agenda-setting and Framing, argue that the news (media agenda) defines for the audience what to think about (the public agenda) and how to think about it (framing public issues). More than 500 published empirical studies have demonstrated that the issues, persons, and topics covered by the news become, to varying degrees, important to public opinion [16]. Survey and laboratory experiments have shown that the framing of news issues significantly affects audiences’ attitudes and public opinion [17], as well as their emotions [18]. A systematic review of longitudinal studies on the duration of news framing effects concluded that effects “persist beyond initial exposure and that may be influential for subsequent decision-making over time, but that the durability of effects heavily depends on whether individuals are exposed to competitive frames also.” [19].

Given that previous studies have already shown that AI is part of the media agenda of the four Chilean newspapers (print press) with the largest circulation in the country [5], this study aimed to identify how the Chilean digital press framed AI before and after the launch of ChatGPT in November 2022. News Framing theory posits that frames are socially shared and persist over time, serving as central organizing ideas or principles for news content. Frames structure the social world by supplying context and determining the issue at stake, selecting and emphasizing certain aspects of reality while minimizing or excluding others [20,21]. This is why frames have the power “to bring otherwise amorphous reality into a meaningful structure, making it more than the simple inclusion or exclusion of information.” [20].

The combined use of sociological and journalistic theoretical perspectives enables a more comprehensive understanding of how AI is represented and interpreted in the media. While sociological approaches illuminate how technologies are socially shaped and embedded in broader imaginaries of the future, journalistic theories such as framing explain how specific narratives are constructed and communicated to the public.

### Press coverage of AI around the world, and this study aims

Press coverage of AI has varied across regions and evolved over time, with notable changes following the launch of ChatGPT, the first AI tool to achieve widespread public use and recognition. Comparative studies reveal persistent disparities between the Global North and Global South in how AI is covered in the media. In both regions, reports most frequently focus on technological trends, business developments, and economic impacts. However, in the Global North, issues including AI regulation, data privacy, and algorithmic bias, while already present in part of media discussions, have received increasing attention since the launch of ChatGPT [2,22–25]. In the Global South, by contrast, these topics have generally remained peripheral over time [2,5,26,27]. For example, while coverage in the Global North has tended to frame generative AI in education as a potential disruptor, highlighting risks to academic integrity and fairness, Chinese media have placed greater emphasis on its role in fostering educational innovation [25]. In Chile, Valderrama et al. [5] reported that, as elsewhere, coverage has been dominated by positive reporting related to industry and economic growth. Still, in recent years, critical perspectives have gained visibility in areas such as the arts, humanities, and law.

The sentiment patterns broadly aligned with these topical differences. In the Global North, sentiments toward AI were generally balanced or optimistic prior to 2023 [28–30], but following the release of ChatGPT, positive sentiment declined and perceptions of risk increased [31,32]. In the Global South, coverage has tended to maintain a predominantly positive tone over the years [27].

This study aims to analyze the coverage of AI in Chilean digital media before and after the launch of ChatGPT, addressing the following specific objectives:

- To identify dominant topics and their associated emotions in AI-related news coverage.
- To identify the key entities referenced in each topic.
- To characterize the frames through which AI is portrayed in the Chilean digital press.

## Data and methods

This study analyzes a total of 1,466 news stories on AI published by six Chilean digital news outlets over periods that begin between 2000 and 2014, depending on the outlet, and extend in all cases until October 19, 2024. This section outlines the steps taken to achieve the stated research objectives.

The data and code used to generate the results are publicly available via a DOI [https://doi.org/10.5281/zenodo.19490096].

### News outlet selection and unit of analysis

We selected a set of news media outlets to represent the online Chilean media system. To identify such outlets, we focused on those with a nationwide reach and significant online presence, as reported in each edition of the Digital News Report from 2017 to 2024 [33]. The Digital News Report analyzes news consumption patterns across various markets, helping establish media prominence and representativeness. Chile was first included in the report in 2017.

Since media outlets meeting these inclusion criteria varied over the five years, we ultimately included the six outlets that maintained a consistent presence across all iterations for the observation period and that were available in the search software Sophia [34]: Bio Bio Chile, La Tercera, Emol, CNN Chile, t13, and Ahora Noticias Mega.

### News collection retrieval

This study employed the search software Sophia [34], a licensed web-based tool for exploring online news media content (https://inf.uach.cl/investigacion/sophia-2/). Sophia enables users to search for and download news content from multiple news media outlets.

A total of 1,466 news items were collected using the keywords “Artificial intelligence” or “AI” (Inteligencia artificial or IA in Spanish). To ensure that only articles covering AI were retrieved, we restricted the search to keywords appearing in the news headlines. Another reason to consider only news headlines to identify stories on AI is that a headline is “the most powerful framing device of the [news] syntactical structure [i.e., headline, lead, episodes, background, and closure]” [35,36].

Since ChatGPT entered the market in late 2022, and to examine its impact on Chilean news coverage, the entire dataset was divided into two subsets (pre-ChatGPT and post-ChatGPT periods) containing news items published before and after January 1, 2023.

### Preprocessing of text, training, and selection of the best LDA topic model

News items in each dataset were automatically pre-processed in Python [37] using the SpaCy library [38]. The cleaning process involved removing stop words, symbols, punctuation marks, and numerical characters. The texts were lowercased and tokenized for normalization [39]. To retain only relevant terms, words with a frequency of occurrence less than 2 (*f* < 2) or present in more than 50% of the documents were excluded [40].

To identify important multi-word expressions, bigrams were constructed and incorporated into the corpus using the Gensim Phrases and Phraser models [41]. Only nouns, adjectives, and verbs were retained for analysis, as these parts of speech are typically the most informative for content analysis.

Subsequently, the LDA algorithm from the Gensim library [41] was applied to train the model using k = 10 topics and the α hyperparameter set to the ‘auto’ function to optimize the best measure for each dataset. To select the model that better fits the dataset, the coherence and perplexity values were calculated for k models (between 2 and 15).

### Topic labelling

In this study, a frame is defined as patterns of specific word associations [42,43] and operationalized as a topic, following the proxy approach suggested by Ylä-Anttila et al. [44].

Topic labeling followed a two-step process. First, the top 30 highest-probability terms per topic were extracted. Second, five representative documents per topic per dataset were automatically selected and independently reviewed by three researchers to identify shared themes and assign a temporal label, following the topic’s interpretation and validation path suggested in previous research [45–47]. Finally, the labels were compared and assigned to each topic.

**Fig 1:**
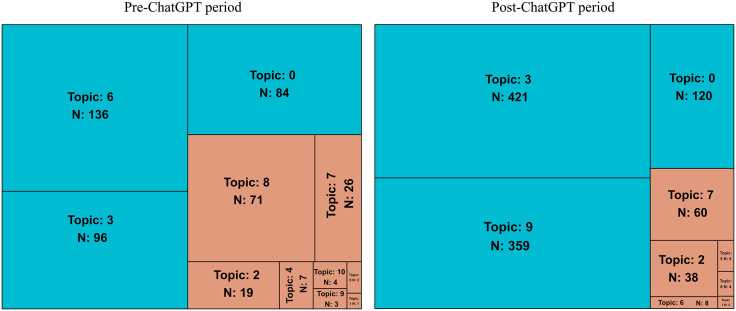
Treemap showing the number, size, and distribution of topics in news on IA in the two periods under analysis. The size of each rectangle is proportional to the number of documents assigned to that topic: in light blue are shown those with the largest number of news items and which were selected for the subsequent analyses (dominant topics).

Given that prominent topics allow for the identification of trends within the dataset and prevent overinterpretation of results, we conducted in-depth analyses for the three most prominent topics per dataset (Fig 1).

To assess whether the dominant topics capture the core components of classical framing theory, a manual validation was conducted on a subsample of representative headlines. Two researchers independently coded four headlines per topic (30 headlines in total) using a structured codebook comprising four frame variables: problem definition, causal attribution, moral evaluation, and proposed solution. Inter-coder reliability was assessed using Krippendorff’s alpha for nominal data, yielding acceptable to solid agreement across all variables (V1: α = 0.84; V2: α = 0.79; V3: α = 0.68; V4: α = 0.67). Disagreements were resolved through consensus discussion. The modal code assigned by human coders for each topic was then compared against the frame components expected from the automated topic descriptions to assess convergent validity.

### Named Entity Recognition (NER)

A Named Entity Recognition (NER) pipeline was applied to identify the main entities present in each topic. Entities were extracted on a per-article basis, keeping only those classified as persons (PER), organizations (ORG), and locations (LOC) for further analyses. To reduce noise, we conducted a cleaning process using a customized stoplist to exclude entities shorter than three characters and lowercase non-proper nouns (e.g., la, el, ya que). Then, entities were normalized to unify aliases into canonical forms (e.g., EE.UU., EEUU, Estados Unidos de América). To reduce duplication and improve the reliability of frequency counts, we applied a heuristic form for personal names: in multi-token entities, only the final token (surname) was retained. Frequency tables were constructed using the retained entities.

Analyses were conducted in Python [37], using pandas [48] for data handling, spaCy [38] and its pre-trained Spanish language model for NER, and the counter class from Python’s collection for frequency aggregation. Visualizations were constructed using the wordcloud package [49] and matplotlib [50].

### Emotion analysis

Emotion analysis was conducted to identify the primary emotions associated with the three dominant topics from each time period using the Quanteda package [51] in R [52]. For this study, the NRC- Emotion Association Lexicon in its Spanish version [53] was employed, focusing exclusively on the eight categorical emotions available: anger, fear, anticipation, trust, surprise, sadness, joy, and disgust.

Lexicon-based analysis was applied at the document level, calculating emotion scores per text based on the frequency of words associated with each emotion. Emotion scores were normalized within each document by converting counts into relative proportions, thereby controlling for differences in document length and variation in the frequency of emotion-bearing words. These scores were aggregated to identify the dominant emotion within each topic. For each dataset, differences in emotion proportions per topic were assessed using pairwise Wilcoxon rank-sum tests with Bonferroni correction to control for multiple comparisons. To evaluate whether the two analytical periods (pre- and post-ChatGPT) differed in their overall emotional content, a permutational multivariate analysis of variance (PERMANOVA) was conducted using the eight emotion scores as a multivariate response. All statistical analyses were conducted using R [52]. Visualizations were constructed using the ggplot2 [54] and treemap [55] packages in R [52].

## Results

A total of 1,466 news items met the inclusion criteria. Media coverage of AI increased considerably from 2023 onwards (post-ChatGPT period), with 69% of the data analyzed coming from the last two years. Two media outlets, Bio Bio Chile and La Tercera, accounted for approximately 55% of all news stories. The number of news items per media outlet is detailed in Supplementary Material S1 in S1 Appendix.

Fig 1 indicates that, in the post-ChatGPT period, fewer topics were identified (nine compared to eleven in the pre-ChatGPT period). This means that although more news items were published in the second period, they were grouped into fewer topics than in the pre-ChatGPT period. Additionally, during the first period, the distribution of news across different topics was more balanced, whereas in the second period, 77% of the news was concentrated in only two topics (topics 3 and 9).

The results of the topic and emotion analyses are summarized in Table 1. A more detailed breakdown of emotions by topic is presented in S1 Appendix.

**Table 1:** Comparison of 3 dominant topics in AI news coverage before and after the launch of ChatGPT.

Dominant topics	Pre-2023 period	Post-ChatGPT period
**1**^**st**^ **most prevalent topic**	*AI Reinterprets Human Identity (Topic 6)*	*Generative AI & Epistemological Uncertainty (Topic 3)*
**Focus**	Creativity, aesthetics, human evolution, authorship	Knowledge limits, security concerns, AI bans
**Dominant emotions**	Joy, surprise, anticipation (↑); fear, trust (↓)	Sadness, anger, surprise (↑); joy, trust (↓)
**Tone**	Innovative, exploratory	Cautious, uncertain
**2**^**nd**^ **most prevalent topic**	*Legislative Adaptation to AI Risks (Topic 3)*	*Acceleration of Medical Advances (Topic 9)*
**Focus**	AI governance, regulation, military/mental health risks	AI in drug development, medical diagnosis
**Dominant emotions**	Fear, trust (↑); anticipation, surprise (↓)	Joy (↑); anticipation (↓)
**Tone**	Urgent, regulatory	Positive, time-oriented
**3**^**rd**^ **most prevalent topic**	*AI-Driven Worker Displacement (Topic 0)*	*Unquestioned AI Progress (Topic 0)*
**Focus**	Task automation, efficiency gains, labor replacement	AI in healthcare, mobility, education — without risks
**Dominant emotions**	Contradictory (Fear & trust (↑); joy, anticipation (↓))	Trust (↑, highest across all periods); anger, disgust (↓)
**Tone**	Contradictory (optimistic + critical)	Uncritical, optimistic

↑ = significantly higher; ↓ = significantly lower relative to other topics in the same period (pairwise Wilcoxon rank-sum tests, Bonferroni corrected).

Manual validation of the six dominant topics yielded an overall agreement of 75% between human coders and the automated analysis (83.3% for problem definition and causal attribution; 66.7% for moral evaluation and proposed solution). Full agreement across all frame components was achieved for four of the six topics, including all three post-ChatGPT topics. Discrepancies were concentrated in the two pre-ChatGPT topics characterized by ambivalent emotional profiles (Legislative adaptation, Topic 3; Worker displacement, Topic 0), consistent with the inherent difficulty of assigning a single dominant frame to topics that combine contradictory tones. These results support the use of dominant topics as proxies for frames.

### Dominant topics and their emotions in pre-ChatGPT AI news coverage

#### AI reinterprets human identity (Topic 6).

This topic highlights how AI reshapes concepts such as creativity, aesthetics, and human evolution. Institutional endorsements (e.g., Royal Academy of Fine Arts, Nature Communications) frame AI as innovative, although controversies have emerged around authorship and regulation. Above all, these institutions highlight the innovative role of AI, producing, for example, never-before-seen fashion, discovering new human species in the fossil record, or producing award-winning works of art.

Emotionally, this topic showed significantly higher levels of joy, surprise, and anticipation, and lower levels of fear and trust than the others in the same period.

#### Legislative adaptation to AI risks (Topic 3).

This topic addresses the urgency expressed by authorities and experts, both from the Chilean State (legislators, ministers) and the scientific community, regarding the need to adapt current legislation to ensure that AI is developed in a controlled and responsible way. Given that AI is expected to have a significant impact on various aspects of life in the near future, it is essential to anticipate the challenges and risks that this technology entails. These issues range from military use to mental health and digital governance.

**Fig 2:**
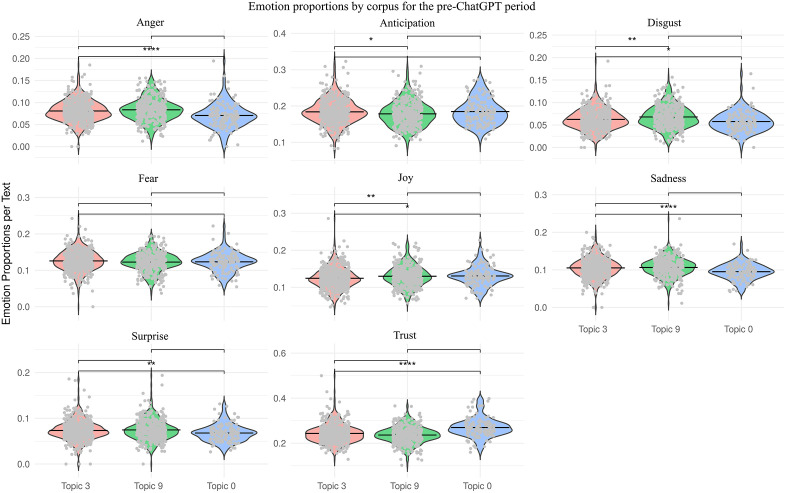
Proportion of emotions in each dominant topic in the pre-ChatGPT period (Y-axis). Significant differences in emotions between topics according to pairwise Wilcoxon rank-sum tests (Bonferroni corrected) are also indicated: * p < 0.05, ** p < 0.01 and **** p < 0.001.

This topic presented significantly higher levels of both fear and trust, along with lower levels of anticipation and surprise, compared to the previous topic (Fig 2).

#### AI-driven worker displacement (Topic 0).

This topic addresses the transformation of activities that were traditionally performed by human workers but are now being replaced or complemented by AI systems. The news emphasizes both the efficiency gains and social limitations of AI in replacing human labor. On the one hand, it highlights the benefits associated with this transition, particularly in the optimization of tasks considered “tedious,” ‘laborious, or “slow.” AI has proven to be able to solve these activities with greater “efficiency” and “speed,” which has generated positive impacts for consumers, companies, and, in some cases, workers themselves.

However, negative effects on AI performance have been observed, particularly in contexts that require a high degree of social sensitivity. One example is the AI’s inability to effectively detect and moderate hate speech and violence in social networks.

Contradictory emotions (positive and negative) are evident in this topic: on the one hand, there is significantly more fear and less joy, while on the other, there is more trust. In addition, there was less anticipation and surprise than for other topics (Fig 2).

### Dominant topics and their emotions in post-ChatGPT AI news coverage

#### Generative AI and epistemological uncertainty (Topic 3).

The generation of new content in areas where human knowledge is still limited raises concerns and precautionary restrictions. This topic does not address everyday problems but rather challenges that significantly affect human beings, states, and large companies. For example, some deep learning models have suggested the existence of extraterrestrial life or predicted the magnitude and location of devastating mega-earthquakes, while scientists remain skeptical, as they do not yet have a definitive answer. Likewise, states such as Italy and companies like Samsung have banned the use of ChatGPT due to security concerns that they do not fully understand and cannot effectively control.

This topic exhibited a marked increase in negative emotions (especially sadness, anger, and surprise) and lower levels of joy and trust compared to other topics during the same period.

#### Acceleration of medical advances (Topic 9).

AI is framed here as a key driver in accelerating medical research and addressing age-related diseases. The time factor is dominant, as it is implicit in the speed with which these scientific advances occur and in the nature of certain medical conditions, such as aging and degenerative diseases. There is a positive assessment of this new pace, which helps to combat aging, reduce normal drug production times, and make earlier diagnoses of degenerative diseases, such as Alzheimer’s and Parkinson’s diseases. This acceleration is often described in concrete terms, such as the reduction in the number of years required for research and pharmaceutical production to bring a drug to market. The actors in this frame are mainly pharmaceutical companies and scientists.

**Fig 3:**
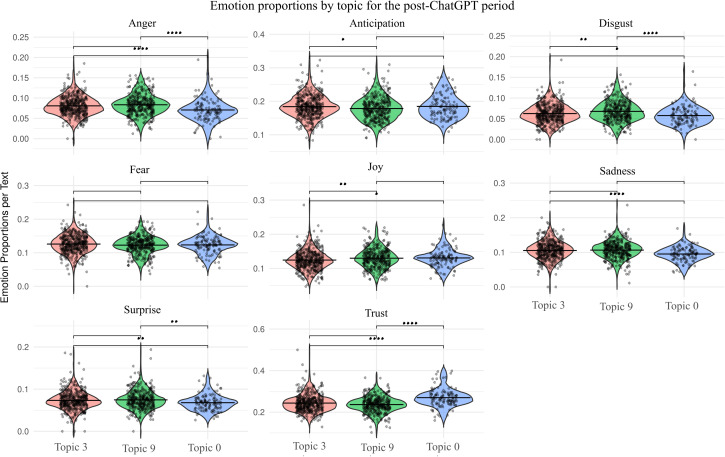
Proportion of emotions in each dominant topic in the post-ChatGPT period (Y-axis). Significant differences in emotions between topics according to pairwise Wilcoxon rank-sum tests (Bonferroni corrected) are also indicated: * p < 0.05, ** p < 0.01 and **** p < 0.001.

This topic exhibits more joy and less anticipation compared to Topic 3 (Fig 3), reflecting its predominantly positive framing. The relatively high levels of anger and disgust observed are likely attributable to the frequent mention of degenerative illnesses in the news coverage, rather than to a negative assessment of AI itself.

#### Unquestioned AI progress (Topic 0).

This topic emphasizes the benefits of AI without referencing the risks or critical perspectives. It features optimistic narratives about public-private cooperation and the societal potential of AI. A narrative of unquestionable progress prevails in several areas: AI optimizes ambulatory care, boosts electromobility, and benefits everything from businesses to educational institutions. Even when mention is made of the need to ensure that it has a positive impact on society, no mention is made of its potential adverse effects.

The importance of establishing public-private partnerships to advance AI implementation is emphasized. The main actors in this topic include representatives of large technology companies (Google, Uber, Microsoft), startups, and actors from the public sphere, such as universities (University of Oxford, Autonomous University) and public services (schools and hospitals).

Emotionally, this topic registered the highest levels of trust across both periods (Figs 2 and 3) and the lowest levels of anger and disgust within its own period (Fig 3).

### Comparative analysis of entity and emotional composition between periods

Emotion analysis showed that each of the six topics was associated with a distinct emotional profile (Figs 2 and 3), and these differences were statistically significant according to the pairwise PERMANOVA test (p < 0.05, Bonferroni corrected). Based on this, we compared both the within-period emotional differences (above) and the broader cross-period trends (here, Table 2).

**Table 2:** Summary of differences in emotional tone between periods.

Emotion	Shift in pattern following the launch of ChatGPT
Anger	Becomes stronger and more uneven
Anticipation	More concentrated in a single topic
Disgust	Emerges as a significantly different factor
Fear	Flattens; less topic-differentiated
Joy	Increases in intensity and specificity
Sadness	Increases in specificity
Surprise	Intensity stable
Trust	Becomes more concentrated and intense

Across all topics, trust was the most prevalent emotion, accounting for approximately 20%–30% of the emotional content in the analyzed news. This was followed by anticipation, which was also frequent but decreased in the second period. In contrast, anger and disgust were consistently the least represented emotions (Figs 2 and 3).

A comparison of the two periods revealed a clear temporal shift in emotional framing (Table 2). While the pre-ChatGPT topics featured more moderate and evenly distributed emotional tones, the post-ChatGPT topics were characterized by greater emotional polarization, particularly in terms of anger, disgust, trust, and joy. For instance, trust grew more concentrated, peaking in narratives that emphasized technological optimism. Similarly, fear, which was central to discussions about legislation and labor (Topics 3 and 0) in the pre-ChatGPT period, became less prominent in the later period. Simultaneously, joy shifted from celebrating AI’s reinterpretation of human creativity to its potential in healthcare and development.

**Fig 4:**
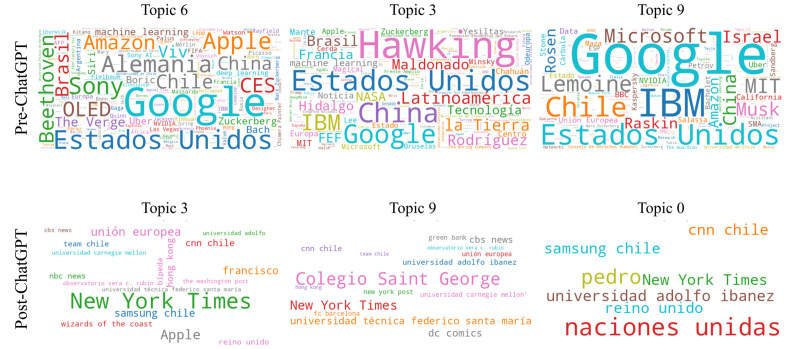
Word cloud indicating the most frequently named entities in the three dominant topics of the pre-ChatGPT and post-ChatGPT periods.

In terms of the actors represented, the pre-ChatGPT period is notable for the overwhelming presence of large technology companies such as Google, Amazon, Apple, IBM, and Microsoft, associated with countries with a strong scientific and technological research ecosystem, such as the US, Germany, China, and, to a lesser extent, Israel, Brazil, and France (Fig 4). These entities are common to the three dominant topics of the pre-ChatGPT period.

In the post-ChatGPT period, the visual field is simplified, less diversified, and fewer actors appear (Fig 4). The focus shifts: in the three dominant topics of this period, there is no longer as much talk about global corporations or states involved in AI’s technological development. Instead, there is a notable appearance of national (Federico Santa María Technical University and Adolfo Ibañez University) and international universities (Carnegie Mellon University) and organizations from different sectors that have implemented AI, such as FC Barcelona, Wizards of the Coast, Team Chile, and DC Comics. The emergence of international press entities (e.g., The New York Times and The Washington Post) is due to certain news items or news segments being reproduced in local media.

## Discussion

This study examined the evolution of public discourse on AI in leading Chilean digital news outlets, asking how the entities, thematic focus, and emotional tone of AI coverage shifted following the public release of ChatGPT in late 2022. By combining computational methods—LDA and emotion analysis—with interpretive approaches grounded in the sociology of technology and framing theory, we identified the dominant topics and emotions characterizing each period and how the actors and frames involved contributed to the construction of a public understanding of AI in the Chilean context. Our findings reveal notable transformations in the topics, entities, and emotions of media discourse following the introduction of ChatGPT.

In the pre-ChatGPT period (2000–2022), coverage was characterized by a complex, multidimensional portrayal of AI, with its efficiency and intelligence acknowledged yet framed within a discourse attentive to potential existential risks (Table 1). These risks were distributed across different domains: labor displacement (Topic 0), state regulation and governance (Topic 3), and human nature and creativity (Topic 6). Temporal orientations varied, spanning reflections on the past, present challenges, and anticipated future risks. Emotionally, these topics combined positive and negative tones, often within the same narrative, reflecting the coexistence of fascination and apprehension (Fig 2).

Post-ChatGPT (2023–2024) coverage revealed a substantial narrowing of topics, entities, and emotions (Table 1). This period revealed fewer actors overall, with attention shifting from the broad presence of global corporations and countries with strong research ecosystems to more concrete organizations where AI is actually implemented, alongside a growing role for universities as central actors in the field (Fig 4). Thematic focus shifted toward concrete applications and benefits, particularly in healthcare (Topic 9) and broader societal progress (Topic 0), while epistemological uncertainty (Topic 3) became the primary locus of negative emotion. Overall, emotion profiles became more polarized, with trust and joy concentrated in specific topics, while fear, which was prominent in the earlier period, diminished (Fig 3; Table 2).

Our results differ from those of Valderrama et al. [5], who reported no significant changes in topics over time (2008–2023) and emphasized the continued dominance of industry hype. A likely explanation lies in the scope of the corpus analyzed. Whereas Valderrama et al. [5] included a smaller set of outlets (four) and broader search terms, our study focused on six prominent online news platforms and on news headlines explicitly referencing AI, which are more likely to shape public perceptions. This approach provides a more targeted perspective on how AI is portrayed in the country’s most influential media outlets.

The transformation in the content on AI aligns with national survey data, which indicate that Chileans increasingly perceive AI as an opportunity rather than a threat [3,4]. It also suggests movement toward the closure of a technological controversy: earlier pluralistic debates over risks and societal implications are giving way to a dominant, optimistic sociotechnical imaginary in which AI’s tangible benefits outweigh its potential harms. This reflects the consolidation of interpretive flexibility [6,9] into a stabilized understanding of AI, supported by institutional alliances that advance this vision (Fig 4).

Following the launch of ChatGPT, the increased news volume, diminished thematic diversity, and sharper emotional contrasts suggest the Chilean press is increasingly aligning around a coherent and optimistic frame for technological progress. We argue that the period following the launch of ChatGPT coincided with a defining moment in shaping both technological rationality and the scientific rationality that underpins it. It was accompanied by the consolidation of an alliance of actors in the public debate (universities and applied-sector organizations) that showcase AI’s diffusion into different domains (sports, culture, entertainment, education), and who, once the efficiency of this technology had been demonstrated, advanced the construction of a dominant sociotechnical imaginary [14,15]. This imaginary predetermines experience and projects the trajectory of transformation across diverse social fields [6,9,56]. This process mirrors the dynamics observed in Canada by Dandurand, McKelvey and Roberge [57], who describe how a convergence of interests among journalists, entrepreneurial experts, and governments can “freeze out” a technology’s controversiality. Frames foreground economic potential and national competitiveness while backgrounding risks, ethical dilemmas, and societal debates, effectively manufacturing a consensus that narrows the scope of public deliberation and marginalizes alternative perspectives. Yet, the marginalization of dissenting or risk-oriented perspectives suggests a narrowing of deliberative space, raising questions about whose visions of the future are being stabilized and whose are excluded [58].

Globally, the Chilean trajectory diverges from the patterns documented in much of the Global North, where the release of ChatGPT has heightened attention to risks such as data privacy, ethical regulation, and algorithmic bias [32,59,60]. In contrast, Chilean media coverage aligns more closely with East Asian contexts, where emphasis is placed on innovation, societal benefits, and collective progress [2,25]. This suggests that imaginaries of AI are not only nationally shaped but also regionally patterned, reflecting broader cultural and political orientations toward science and technology. In Chile’s case, this may resonate with its broader sociocultural trajectory, as survey evidence highlights a greater affinity with East and South Asian orientations, characterized by secular values, strong faith in science and technology, and a prioritization of collective well-being over individual self-expression [61,62].

Here, we demonstrate how the Chilean press has mediated a shift in the public framing of AI, from emphasizing its development to highlighting its implementation. In this latter phase, reporting adopts a markedly positive tone and draws on a narrower set of voices, which has contributed to settling much of the technological controversy surrounding AI. Rather than merely mirroring technological change, the press actively structured the debate by amplifying some topics, emotions, and actors while sidelining others, shaping not only which expectations gained traction but also how they coalesced into a relatively stable, opportunity-oriented sociotechnical imaginary.

The period following the release of ChatGPT marked, then, a turning point in Chilean AI discourse, coinciding with the closure of earlier debates about risks and the consolidation of a techno-optimistic imaginary. The coverage of digital news outlets shifted from pluralistic, emotionally balanced discussions to a narrower and more concrete focus on progress, predominantly framed in positive terms. We interpret this shift as a movement toward the closure of a technological controversy, in which a dominant socio-technical imaginary (shaped by alliances between universities and applied-sector organizations that showcase AI’s diffusion into different domains) frames AI as an inevitable and beneficial force. While this reflects broader societal acceptance of AI, it also risks sidelining critical voices, a pattern that warrants scrutiny as Chile navigates the ethical and social dimensions of AI integration.

### Limitations and future research

A limitation concerns the emotion analysis tool employed. The Spanish version of the NRC Emotion Lexicon was generated by automatically translating the original English terms, rather than being independently validated for Spanish-speaking populations. While the assumption of cross-linguistic affective stability has been broadly supported in the literature, the lexicon was not specifically validated for Chilean Spanish. It should be noted, however, that the corpus analyzed consists of news articles from national digital media outlets, which tend to use formal, standardized written Spanish rather than colloquial or region-specific expressions. This register likely reduces the impact of dialectal variation on emotion scores. Nonetheless, future research would benefit from the development or use of sentiment resources specifically adapted to Latin American Spanish varieties.

Although our corpus includes news from outlets with diverse ownership, political ideologies, and target audiences (both elite and popular), the analysis treated all articles as if they originated from a single source. This decision reflects the study’s objective of characterizing the overall information environment that Chilean audiences are actually exposed to, rather than explaining outlet-specific editorial choices. Several directions for future research follow from this and other limitations. How ownership, ideology, and audience differences shape AI framing remains an important open question, as does the broader challenge of tracking which values, actors, issues, and emotions are currently relevant and which are becoming obsolete. We are at a unique moment of technological implementation, making longitudinal studies particularly valuable. Future work could also include comparative analyses of Chile’s trajectory with other Global South nations to identify regional patterns, outlet-level studies to assess the influence of editorial policies, and ethnographic approaches to explore how public perception of AI is formed and evolves.

## Supporting information

S1 Appendix Radar plots of sentiment distribution by topic. (PDF)
