# Supplementary material for: Framing artificial intelligence in Chilean digital press before and after the launch of ChatGPT: From concern to optimism
Source: PLoS One. 2026 May 11;21(5):e0348680. doi: 10.1371/journal.pone.0348680 (PMC13160352; doi:10.1371/journal.pone.0348680)
Supplement: S1 Appendix — (PDF) [file pone.0348680.s001.pdf]

Supplementary Material 1. Radar plots of sentiment distribution by topic.

This supplementary material presents radar plots for each of the six topics identified in the article through topic modeling. Each plot displays the proportion of sentiments represented within a given topic, allowing for visual comparison of affective profiles across topics. Values range from a minimum of 0 to a maximum of 1, where 1 indicates that all articles associated with a topic carry that particular sentiment. Plots are provided separately for the pre-ChatGPT and post-ChatGPT periods, reflecting the temporal structure of the analysis presented in the main text.

Radar Plot: Emotion Distribution Across the Entire Corpus of Topic 6 (pre-ChatGPT)

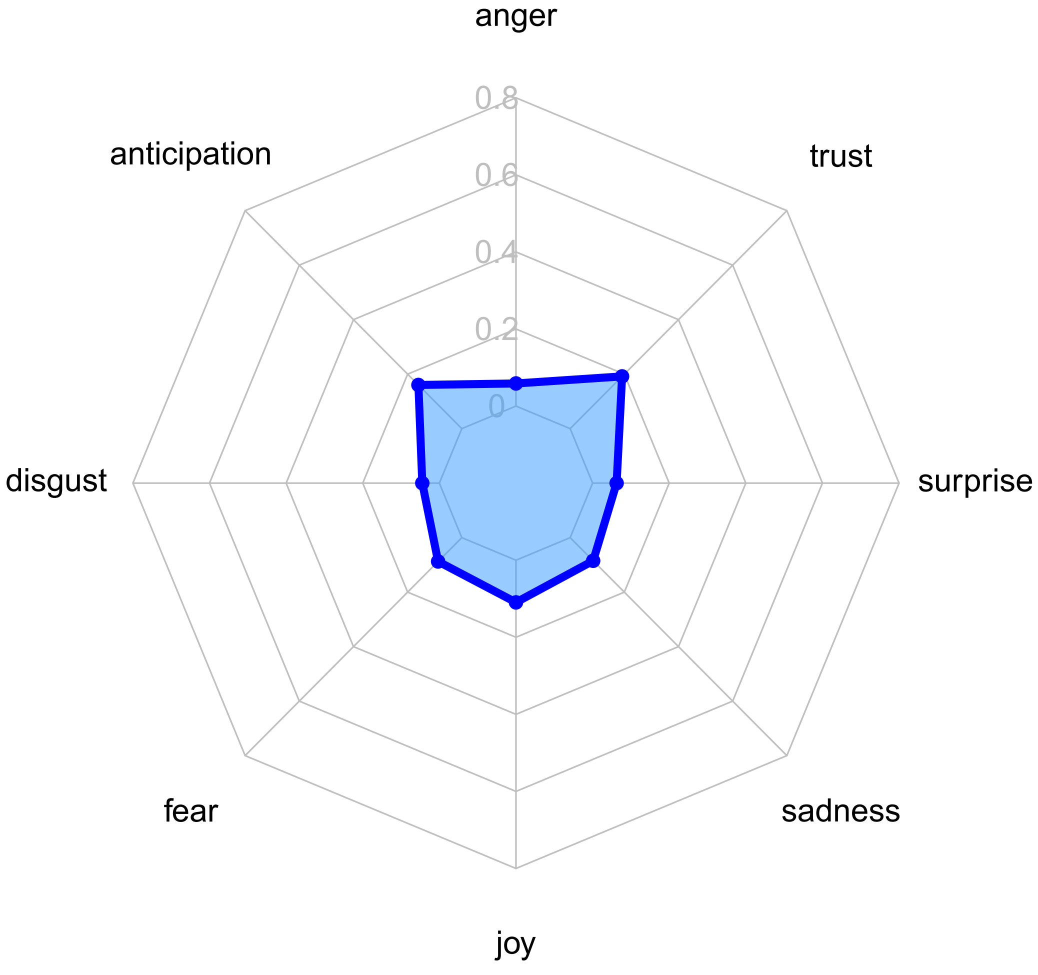

Radar Plot: Emotion Distribution Across the Entire Corpus of Topic 3 (pre-ChatGPT)

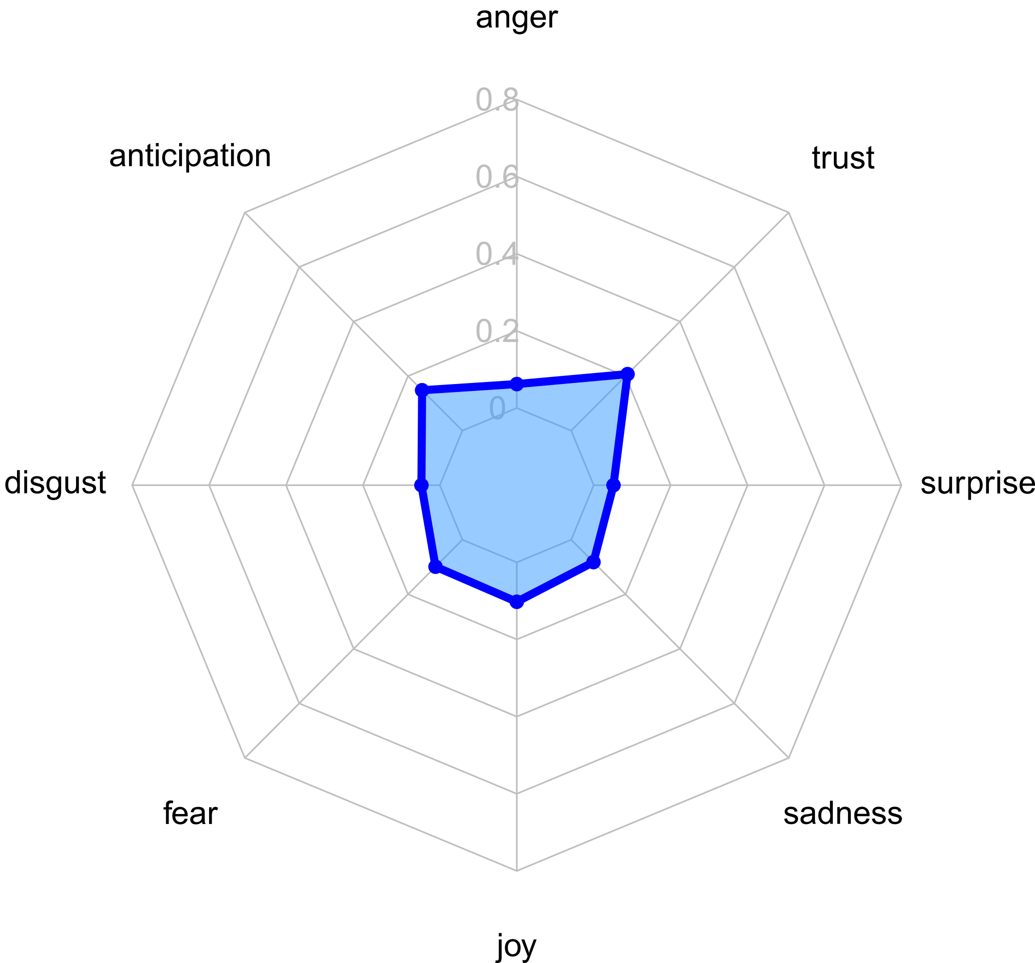

Radar Plot: Emotion Distribution Across the Entire Corpus of Topic 0 (pre-ChatGPT)

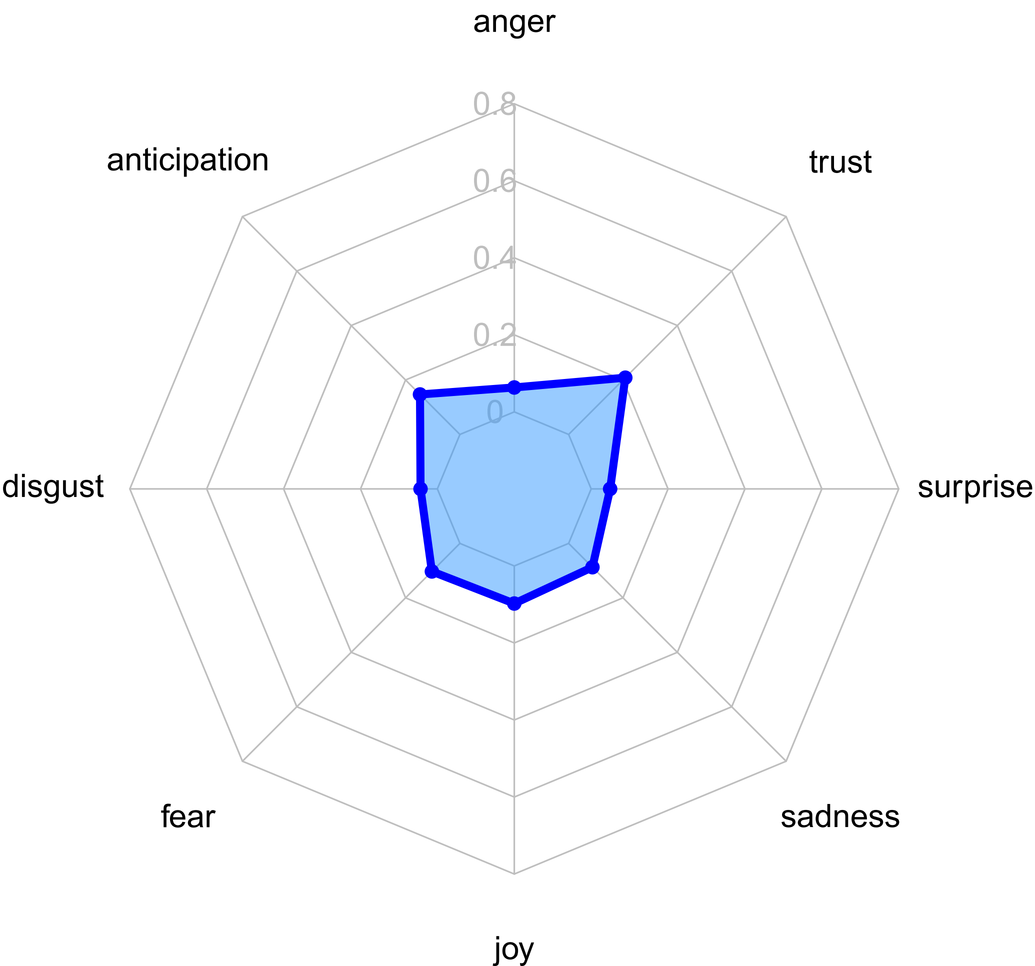

Radar Plot: Emotion Distribution Across the Entire Corpus of Topic 3 (post-ChatGPT)

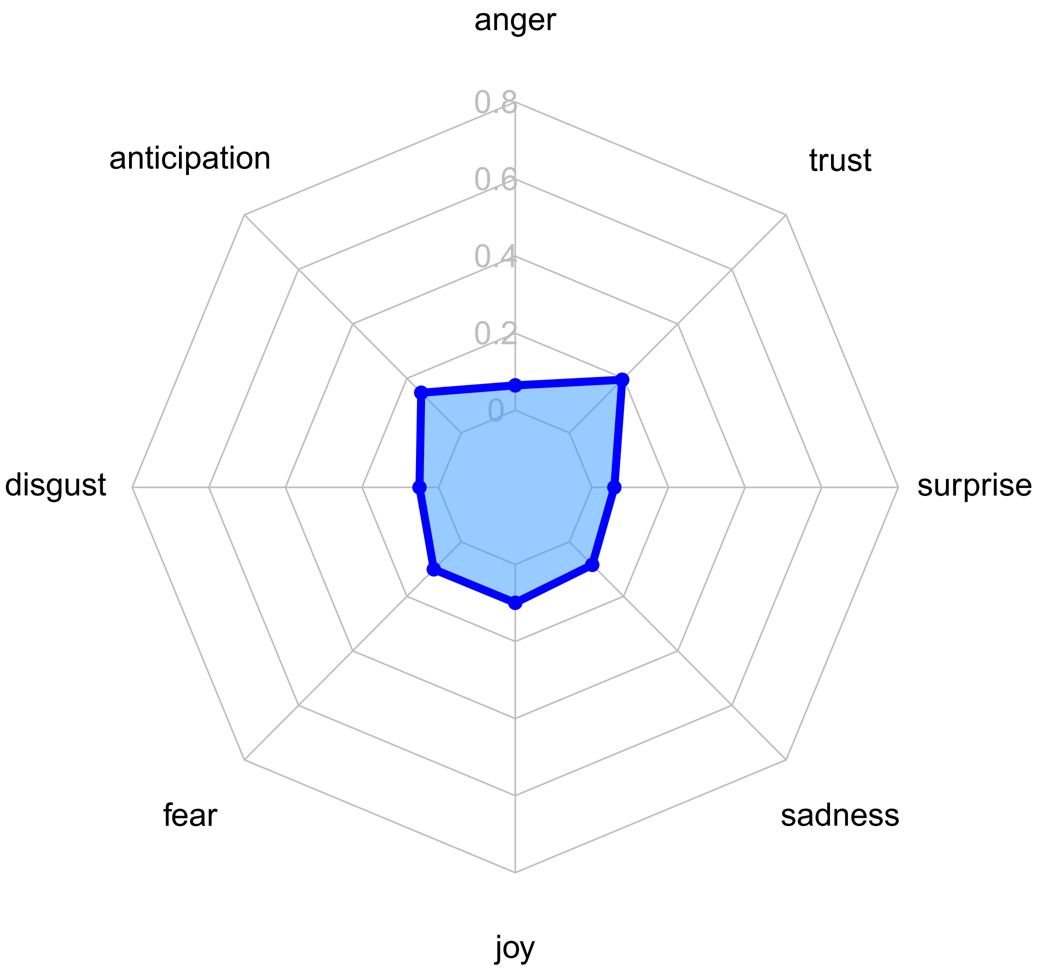

Radar Plot: Emotion Distribution Across the Entire Corpus of Topic 9 (post-ChatGPT)

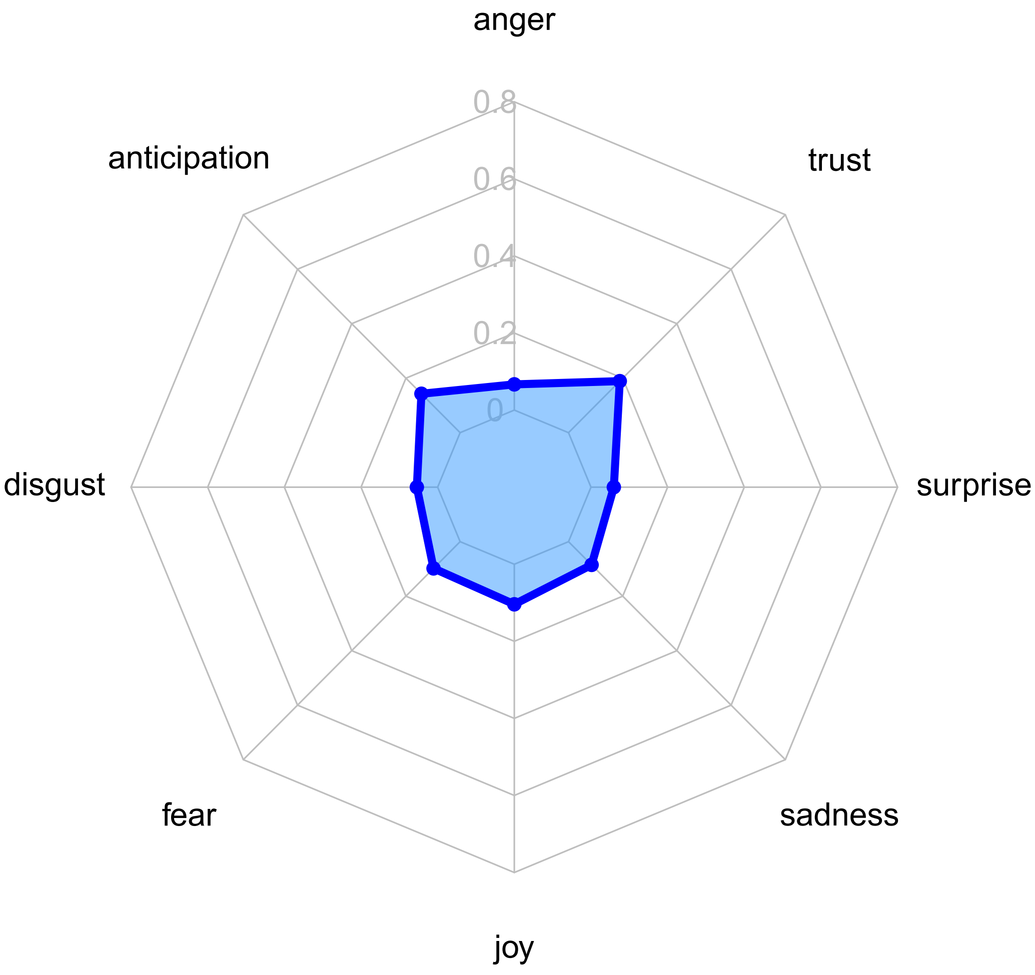

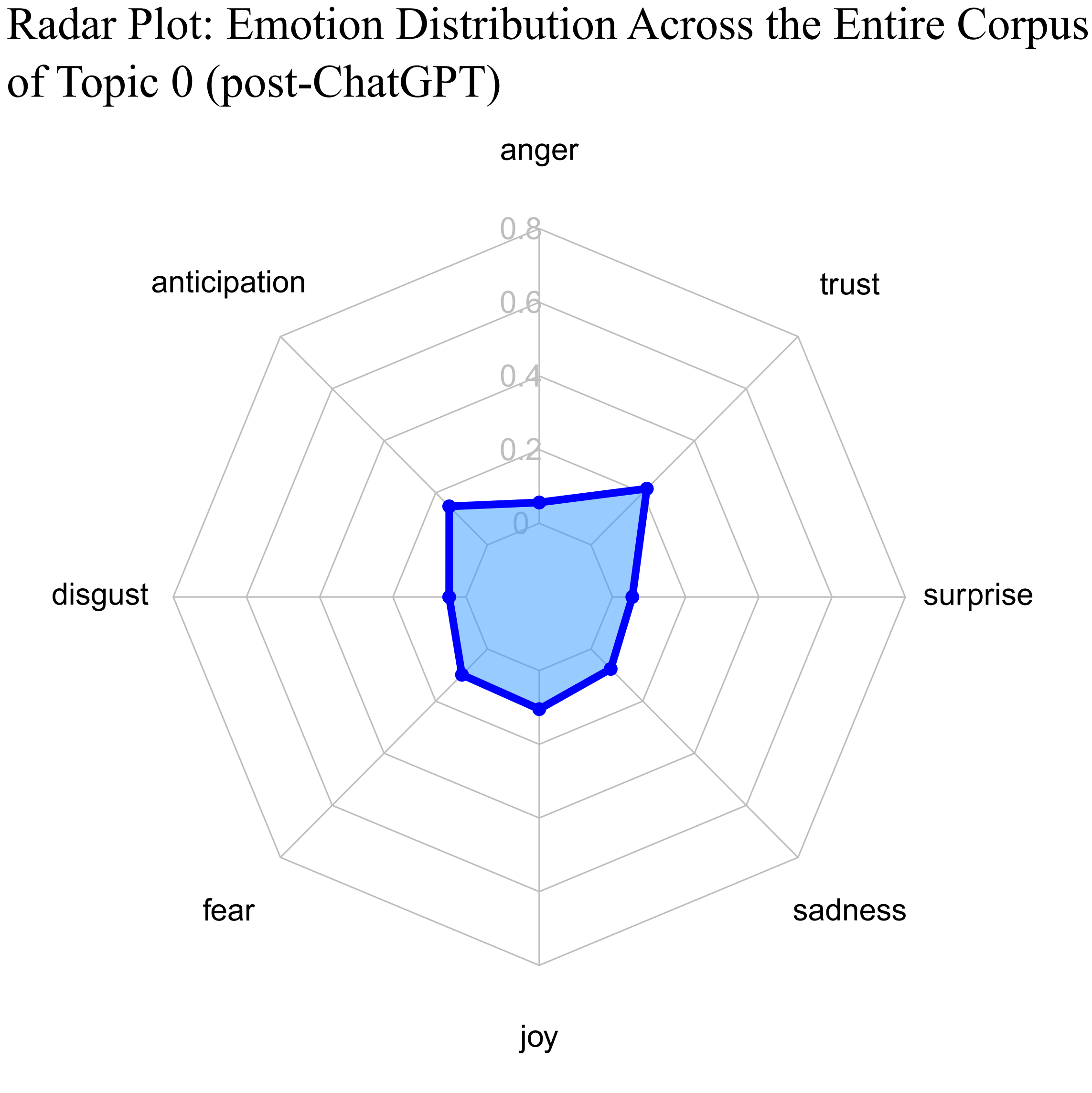

Radar Plot: Emotion Distribution Across the Entire Corpus of Topic 0 (post-ChatGPT)
anger
trust
surprise
sadness
joy
fear
disgust
anticipation
0.8
0.6
0.4
0.2
0
